# Nonverbal Communication Processing in Deaf Adults: An Activation Likelihood Estimation Meta-Analysis

**DOI:** 10.3390/brainsci15121299

**Published:** 2025-11-30

**Authors:** Shimin Mo, Andrew Dimitrijevic, Claude Alain

**Affiliations:** 1 Institute of Medical Science, Temerty Faculty of Medicine, University of Toronto, Toronto, ON M5S 1A8, Canada; andrew.dimitrijevic@sunnybrook.ca (A.D.); calain@research.baycrest.org (C.A.); 2 Evaluative Clinical Sciences Platform, Sunnybrook Research Institute, Toronto, ON M4N 3M5, Canada; 3 Rotman Research Institute, Baycrest, Toronto, ON M6A 2E1, Canada; 4 Department of Otolaryngology-Head and Neck Surgery, Temerty Faculty of Medicine, University of Toronto, Toronto, ON M5S 1A1, Canada; 5 Department of Psychology, University of Toronto, Toronto, ON M5S 3G3, Canada; 6 Music and Health Research Collaboratory, Faculty of Music, University of Toronto, Toronto, ON M5S 2C5, Canada

**Keywords:** hearing loss, deafness, neuroplasticity, nonverbal communication, neuroimaging, meta-analysis, activation likelihood estimation

## Abstract

**Background/Objectives**: Hearing loss affects spoken language processing and leads to cortical reorganization in sensory systems. While neuroimaging research has explored cross-modal plasticity in visual language processing, there is a need to identify brain activation patterns consistently activated across different nonverbal communication tasks in deaf individuals. We hypothesized that deaf adults would show convergent activation across studies in visual and auditory cortices during nonverbal communication processing compared to typical hearing adults. **Methods**: To test this, we conducted an Activation Likelihood Estimation analysis of 14 neuroimaging studies using different visual linguistic stimuli and tasks in adults with prelingual deafness and age-matched hearing controls. **Results**: Contrary to expectations, deaf individuals did not show intramodal activation in the visual cortex. Instead, they demonstrated convergence activation in the left superior temporal gyrus only, indicating cross-modal recruitment of auditory regions, which supports visual-spatial language processing. **Conclusions**: These findings highlight the need for future research to clarify how cortical reorganization impacts speech perception outcomes following auditory restoration using neuroprostheses like cochlear implants.

## 1. Introduction

The human brain can exhibit neuroplastic changes, an adaptive ability that, in many contexts, allows it to respond and adjust to diverse environmental factors and experiences by modifying the functional and structural organization of the nervous system [1]. Deaf individuals experience auditory deprivation. While devices such as cochlear implants and hearing aids can enhance hearing, their technical constraints, such as limited temporal and spectral resolution [2], prevent them from having fully restored hearing. Hence, deaf individuals often adaptively rely on nonverbal communication, such as sign language and speechreading, to compensate for reduced auditory input when navigating daily life. Understanding how deaf individuals rely on these visual linguistic systems is essential for revealing how the brain reorganizes in response to auditory deprivation, setting the foundation for investigating the neural mechanisms that support nonverbal communication.

### 1.1. Neural Correlates of Different Types of Nonverbal Communications

For this study, we define nonverbal communication as linguistic stimuli presented in the visual domain, including sign language, speechreading/lipreading, and written language. Speechreading involves interpreting spoken language by observing facial expressions, lip movements, and tongue positioning [3]. In contrast, sign languages such as American Sign Language (ASL) and British Sign Language (BSL) are visuospatial and incorporate both manual gestures and non-manual signals (e.g., facial expression, mouth movements) that disambiguate lexical meaning [4].

Although speechreading and sign language differ in articulatory features (lip movements versus hand gestures), they share important linguistic and neural properties [4]. In sign languages, the mouth provides adverbial, grammatical, and phonological information and can serve as a minimal unit distinguishing lexical items. Some manual signs involve mouth movements that mirror hand actions, suggesting that both channels may support similar perceptual and linguistic processes.

A substantial body of neuroimaging evidence shows that the brain processes sign and spoken language using a shared left-lateralized perisylvian network, independent of modality. fMRI studies by Emmorey and colleagues [5] and by MacSweeney and colleagues [6,7,8] demonstrate that sign language comprehension engages the inferior frontal gyrus (IFG), superior temporal gyrus (STG), and inferior parietal lobule (IPL). Additional findings reveal enhanced left-lateralized activation in the ventral middle frontal gyrus (MFG) and IFG during the semantic-level processing of sign language [9]. Silent speechreading in deaf adults elicits robust activation in the left STG, including auditory-associated regions, compared to hearing adults, indicating preferential access of speech-like visual actions to auditory speech cortex [4].

Written words, although lacking dynamic visuospatial motion, also activate language-related auditory regions in deaf individuals. During visual, semantic, and phonological judgement tasks, deaf readers recruit the planum temporale, left IFG, middle temporal gyrus (MTG), and STG—the same regions engaged during sign language and speechreading [10,11,12]. Thus, sign language, speechreading, and written words share overlapping neural circuits and are considered collectively as nonverbal communication modalities in this meta-analysis.

Together, these findings suggest that visual linguistic systems place substantial demands on the visual and language networks. To understand how such demands reshape the brain, it is essential to examine the forms of cortical reorganization that arise from auditory deprivation.

### 1.2. Intramodal Plasticity

Intramodal plasticity strengthens intact cortical regions within the same sensory modality [13]. In deaf individuals, visual intramodal reorganization may emerge due to increased reliance on visual input. Childhood-onset deafness is associated with enhanced visual abilities, including greater peripheral visual attention [14], improved visual change detection [15], heightened visual motion sensitivity [16], and faster visual responses [17,18]. These enhancements are thought to arise from reorganization of the visual cortex in response to reduced auditory input [19].

Such intramodal changes may persist even after auditory sensation is restored through CIs. Doucet et al. [20] examined visual motion–evoked responses in early-deaf CI users and found that individuals with higher speech perception scores exhibited stronger occipital P2 waveforms around 200 ms. The authors interpreted this as evidence of intramodal visual reorganization and audiovisual coupling associated with successful post-implant speech perception.

In summary, auditory deprivation may lead to heightened intramodal visual processing in deaf adults, particularly during nonverbal communication tasks such as sign language comprehension and speechreading.

### 1.3. Cross-Modal Plasticity

For people with hearing loss, another type of neuroplasticity, cross-modal plasticity, can also occur because of auditory deprivation and reduced auditory input. Cross-modal plasticity refers to the ability of an intact sensory system, such as vision or somatosensation, to recruit cortical regions typically associated with another sensory modality (e.g., audition) to support sensory processing. Since deaf individuals primarily rely on non-verbal communications like lipreading or sign language for daily interactions, visual take-over, the activation of the auditory cortex in response to visual stimuli, is often observed [21]. Importantly, the auditory cortex is where the spoken language is conventionally processed, especially for segmenting phonemes within a word-for-word discrimination and further semantic processing [22].

Auditory cortical areas are activated for visual processing in pre-lingually and post-lingually deafened adults and adults receiving CIs [23,24,25,26,27,28]. For example, a study by Campbell and colleagues examined cortical cross-modal activity in adults with various degrees of hearing loss [29]. They recorded visual evoked potentials (VEPs) in response to a visual-motion stimulus in individuals with bilateral, early-stage, mild-moderate hearing loss and an age-matched sample of adults with typical hearing. The hearing loss group showed recruitment of the auditory cortex for the visually evoked responses peaking about 170 and 250 ms post-stimulus. These responses were greater in amplitude and peaked earlier in those with hearing loss than in their counterparts with typical hearing. Additionally, source analyses of the VEP N1 and P2 deflections revealed a greater activation along the ventral visual stream in temporal areas related to auditory processing in people with hearing loss compared to typical hearing adults. Similarly, pre-lingual and post-lingual deafened CI users showed an N1 VEP over the right temporal lobe in response to visual motion stimuli. In contrast, the topographic response of hearing controls to the same visual stimuli primarily peaked over the left parietal and occipital lobes [23]. People with congenital deafness also show cross-modal plasticity in the auditory cortex [30]. A group of early-deaf adults (age of onset of deafness < 2 years) performed a visual-spatial working memory task while functional magnetic resonance imaging (fMRI) data were collected. Deaf participants exhibited higher activation levels in auditory association regions than hearing participants, both when complex visual stimuli were presented and during the maintenance phase, when only a static crosshair was visible on the screen.

Additionally, studies using voxel-based and surface-based morphometry show alterations in neuroanatomical structures in people with hearing loss. Deaf signers had reduced white matter underneath left Heschl’s gyrus and in the right posterior middle frontal gyrus when compared to hearing signers and hearing non-signers [31]. A recent study found increased cortical thickness in the left inferior parietal gyrus and right superior parietal gyrus in participants with noise-induced hearing loss relative to controls, consistent with compensatory enhancements in visual and language processing associated with reduced auditory input [32]. Importantly, those with hearing loss also had increased surface area and cortical volume in the left lateral occipital cortex compared to controls [32], which may be a direct result of cross-modal plasticity. Similar structural reorganization has been observed in individuals with tinnitus, where aberrant auditory input is thought to trigger cortical and grey matter changes in auditory [33,34] and non-auditory regions [35,36], further highlighting the neuroplastic consequences of auditory deprivation.

Is cross-modal plasticity reversible when auditory sensation is restored? The progression of cortical reorganization from the initial phases of restoration of auditory function through CI remains an open question. Given that cross-modal plasticity can occur in individuals with various degrees of hearing loss, it is critical to understand whether such reorganization supports or hinders auditory rehabilitation. Current literature suggests two competing perspectives [19]. The “flexibility view” posits that individuals with auditory deprivation may benefit from CI due to the brain’s inherent capacity for plasticity. The opposite “limited capacity view” argues that neuroplastic changes in adulthood are limited, and that extensive reorganization during deafness, especially cross-modal recruitment, may hinder adaptation to novel auditory input provided by the CI. According to this view, such reorganization may be maladaptive and negatively impact CI outcomes.

Building on these theoretical perspectives, recent empirical findings have shed light on the potential consequences of cross-modal plasticity [25,37]. Although additional longitudinal studies incorporating pre- and post-implantation comparisons are needed to clearly distinguish adaptive versus maladaptive patterns of cortical reorganization, existing research has provided indirect evidence linking neuroplasticity and speech perception outcomes in individuals with hearing loss and with CI [25,37]. Whether cross-modal reorganization is beneficial may depend on the type of visual stimulus. A study using low-level stimuli like checkerboards and simple shapes found a negative correlation between cross-modal activation in the auditory cortex and verbal speech recognition abilities in CI users [25]. This indicates a visual takeover in the auditory cortex of individuals who had hearing loss but regained partial hearing through CI, and greater cross-modal activation is related to worse speech intelligibility.

In a different study, cross-modal activation in response to nonverbal communication (presented through lip-reading videos) was positively correlated with better speech understanding in CI users [37]. Participants with CI performed lip-reading while undergoing functional near-infrared spectroscopy (fNIR) scanning. Compared to hearing individuals, those who showed greater cross-modal activation in the auditory cortex from before to after implantation also showed better speech perception abilities. Similarly, a study using electroencephalography (EEG) to measure brain regions activated by lip-reading of single-syllable words in CI users also found that stronger cross-modal activation response in the auditory cortex was associated with higher speech scores compared to hearing individuals [26]. In summary, deaf individuals may show greater cross-modal activation in the auditory cortex in response to nonverbal communication than hearing individuals.

### 1.4. Current Study

Despite decades of research, no quantitative synthesis has identified which brain regions consistently support nonverbal communication (sign language, speechreading, and reading) in deaf adults. Existing reviews focus on a single modality (e.g., sign language only [38]) and lack a comparison with hearing participants. An Activation Likelihood Estimation (ALE) meta-analytic approach is therefore needed to determine convergent patterns of neural reorganization.

This meta-analysis combined neuroimaging studies from the past 22 years, using ALE to identify brain areas consistently associated with nonverbal communication in deaf adults. The primary goal is to ascertain the shared neural network enabling nonverbal communication in people with deafness, shedding light on the reorganization of the brain network following auditory deprivation. We hypothesized that deaf adults would show convergence of activation across studies in visual and auditory cortices during nonverbal communication processing compared to hearing adults, reflecting intra- and cross-modal plasticity. Recognizing the neuroplasticity within the early deaf population helps inform more effective rehabilitation strategies for this population, ultimately enhancing speech perception outcomes. Given the positive correlation between cross-modal activation in the auditory cortex and speech perception performance [26,37], the results of this study could also be used to compare with previous findings and ascertain whether nonverbal communication activates the same brain areas that are positively correlated with CI outcomes.

## 2. Method

This meta-analysis adhered to the PRISMA 2020 (Preferred Reporting Items for Systematic Reviews and Meta-Analyses) guidelines [39], although it was not preregistered.

### 2.1. Search Strategy

A search was conducted in PubMed, Medline, and ProQuest using the following terms: “hearing loss”, “deafness”, “visual language perception”, “lip reading”, “neuroplasticity”, “brain reorganization”, crossed with “fMRI”, “PET”, “functional magnetic resonance imaging”, “positron emission tomography”, and “neuroimaging”. The search included studies published in English peer-reviewed journals as of August 2025.

### 2.2. Screening Process

**Figure 1:**
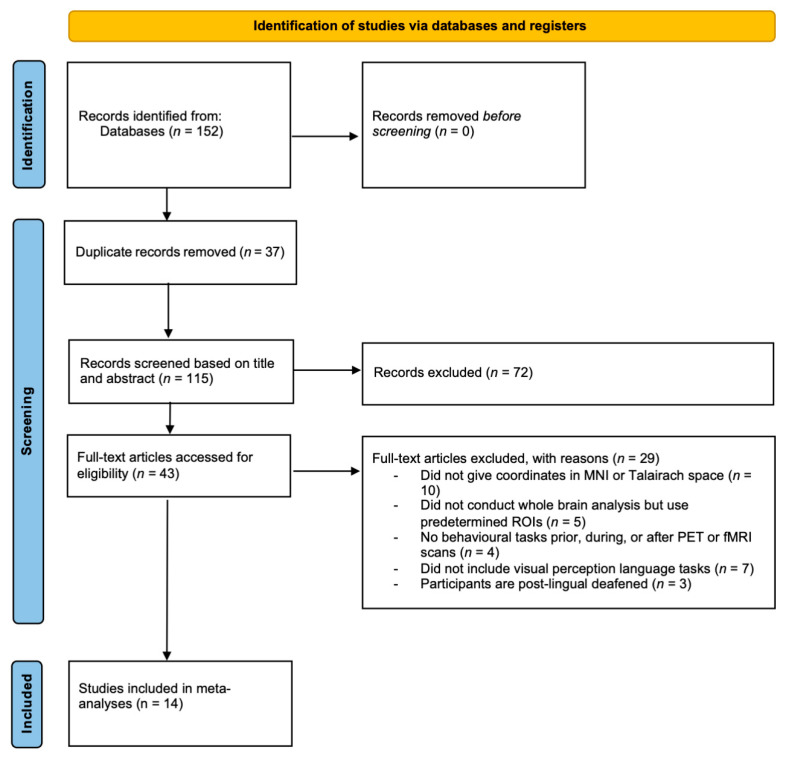
Study flowchart according to PRISMA 2020.

The title and abstract of each record were screened based on PRISMA 2020 guidelines [39]. The full texts of potentially eligible articles were then retrieved and screened. Figure 1 displays the complete screening process. PRISMA checklist is shown in the Supplementary Materials.

Articles were included if they met the following criteria: (a) language-related materials are presented visually (low-level stimuli like checkerboards and simple shapes were excluded); (b) a behavioural task performed during the scanning; (c) a control or baseline condition; (d) whole-brain analysis from fMRI or positron emission tomography (PET) on 3D coordinates in either Talairach [40] or Montreal Neurological Institute (MNI) standardized space; (e) deaf adults, and typical hearing people, all of which had no psychiatric or neurological disorders, or brain abnormalities (e.g., stroke, or tumour); (f) deaf participants were congenitally or prelingually deafened. Studies meeting these selection criteria used a variety of stimuli and tasks, such as speechreading tasks, phonological processing tasks, and sign language judgement tasks. Additionally, since hearing loss could occur congenitally, this paper only focuses on adult participants, as the developmental period (around three to four years old) during which intrinsic and extrinsic inputs refine the auditory system [41] could add more confounders to the analysis.

Although deaf-participant data can, in principle, be compared against large-scale hearing databases (e.g., in [38]), our meta-analysis intentionally included only studies that provided their own hearing control group. This methodological choice ensured that comparisons were made within the same experimental framework, allowing us to conduct the following: (1) Control for study-specific methodological differences (e.g., task design, imaging parameters, analysis pipelines) by relying on contrasts reported within the same experimental context. (2) Minimize heterogeneity between datasets, since results from studies without hearing controls would have required indirect comparisons across different cohorts and tasks. (3) Focus the scope of our analysis on direct group differences in brain activation between deaf and hearing participants, which was the central question motivating our study. We recognize that incorporating all available deaf-participant studies and modelling them against external hearing datasets is a valuable complementary strategy. However, to maximize methodological rigour and internal validity, we prioritized studies with explicit within-study control groups. Although this decision limited the number of eligible studies, it enhances the reliability of our findings. Notably, many ALE meta-analyses comparing clinical groups to controls adopt the same criterion [42,43,44,45,46]. For simplicity, normal-hearing people are referred to as hearing participants.

### 2.3. Activation Likelihood Estimation

Coordinate-based quantitative meta-analyses of neuroimaging results were performed using the GingerALE software (version 3.0.2) available on the BrainMap website (http://brainmap.org/ale/index.html, (accessed on 25 August 2025)). Talairach coordinates were converted to MNI space using the nonlinear registration [47] before being entered into the analysis. Based on coordinates from the included articles, GingerALE generates a brain activation map and applies a permutation test to assess the statistical reliability of the group mean activation. The foci were translated into spatial probability distributions with a width determined by empirical estimates of spatial uncertainty resulting from inter-template and inter-subject variability. These distributions are centred at the specified coordinates. Finally, a random-effect inference was drawn by comparing the ALE results with the null distribution of the random spatial association across studies [48].

We gathered foci and built two datasets, the first one with the coordinates of the peak coordinates where deaf participants showed activation from a direct comparison of the nonverbal communication tasks with a control task (e.g., speechreading versus baseline), and the other one with peak coordinates where hearing participants showed activation from a direct comparison of the nonverbal communication tasks with a control task. Next, cluster-wise scores were computed to show convergence in brain regions across studies. We applied thresholds of *p* < 0.001 for cluster-level FWE (cluster forming threshold) and *p* < 0.05 for cluster-level family-wise error, with 1000 thresholding permutations, following recommendations [49]. We used a more conservative grey matter mask and applied the Turkeltaub nonadditive random-effects method, which reduces both within-experiment and within-group effects by limiting the probability values of neighbouring foci originating from the same experiment [50].

A subsequent ALE analysis contrasted the spatial convergence of activation foci between deaf and hearing participants during visually presented stimuli, generating contrast maps highlighting regions more consistently recruited in deaf than hearing participants. In GingerALE, contrasts were computed using a cluster-level minimum statistic [51], identifying the intersection point where separately thresholded meta-analysis results intersect. This process generated a new thresholded (*p* < 0.05) activation likelihood estimation (ALE) image and facilitated cluster analysis. For the contrast, we used an uncorrected *p*-value of 0.05, 10,000 permutations and a minimum volume threshold of 100 mm^3^. Two-dimensional cluster ALE images and 3D images were created using MRIcroGL (https://www.mccauslandcenter.sc.edu/mricrogl/ (accessed on 28 August 2025).

### 2.4. Jackknife Sensitivity Analysis

We conducted a Jackknife sensitivity analysis to evaluate the robustness of the ALE results against potentially spurious effects like publication bias in the literature [52]. This procedure entailed conducting 14 separate meta-analyses, each excluding one experiment from the original deaf participant dataset. We then visually inspected the extent to which each simulation replicated the original results in terms of the number, location, and size of significant ALE clusters.

## 3. Results

A thorough exploration across all databases identified 149 studies, as depicted in the PRISMA flowchart [33] in Figure 1. Following this screening process, 14 papers were deemed suitable for coordinate-based meta-analysis, encompassing 177 deaf participants and 192 hearing participants. Table 1 provides information on the age and the number of deaf and hearing participants in each study. All deaf participants had prelingual or congenital hearing loss. In studies involving sign language, deaf participants had acquired proficiency in sign language beforehand. Foci used in the ALE analysis are available in the Supplementary Materials.

### 3.1. Neural Substrates of Nonverbal Communication in Deaf Individuals

**Figure 2:**
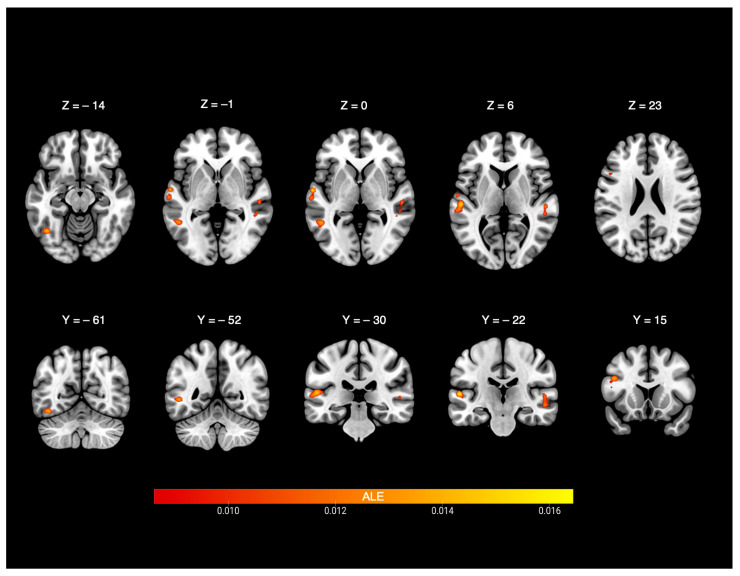
ALE-statistic maps of nonverbal communication perception in deaf people. ALE = Activation Likelihood Estimation.

The analysis included 191 foci from deaf participants and yielded five significant clusters (see Table 2 and Figure 2). The first cluster was centred at the left STG (−57.2, −22.5, 6). This cluster had peak activities in the left hemisphere and involved the following gyri: STG (85.4% of contributed foci), transverse temporal gyrus (11.5%), and MTG (3.1%). The second cluster was centred at the right STG (53, −30, −0.7). This cluster showed peak activities in the right hemisphere and involved the following gyri: STG (94.7%) and MTG (5.3%). The third cluster was centred at left IFG (−45.9, 15.4, 23.2). This cluster had peak activities in the left hemisphere and involved IFG (80.8%) and MFG (19.2%). The fourth cluster was centred at left MTG (−51.7, −51.5, 0.4). This cluster had peak activities in the left MTG only. The fifth cluster was centred at the left fusiform gyrus (−42.8, −61.1, −13.3) and involved peak activity in the fusiform gyrus (58.8%) and declive (41.2%).

### 3.2. Neural Substrates of Nonverbal Communication in Hearing Individuals

**Figure 3:**
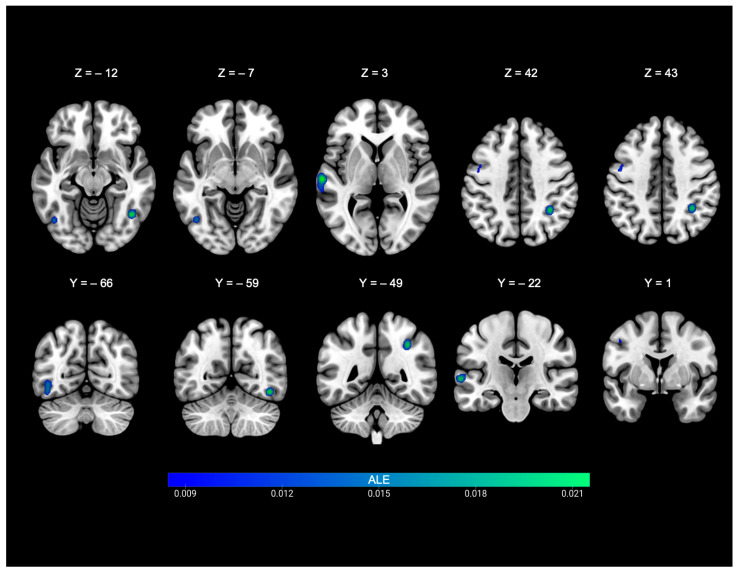
ALE-statistic maps of nonverbal communication in hearing individuals. ALE = Activation Likelihood Estimation.

The analysis included 156 foci from hearing participants and yielded five significant clusters (Table 3 and Figure 3). The first cluster was centred at the left STG (−63.5, −21.4, 2.9). This cluster had peak activities in the left hemisphere and involved the following gyri: STG (73.4%) and MTG (26.6%). The second cluster was centred at the left precentral gyrus (−46.1, 0.6, 42.2). This cluster showed peak activities in the left hemisphere and involved the precentral gyrus (83.1%) and MFG (16.9%). The third was cluster centred at the right IPL (34.2, −48.6, 41.7). It showed peak activities in the right hemisphere and involved the IPL (50%) and angular gyrus (50%). The fourth cluster was centred at the left inferior occipital gyrus (IOG, −44.7, −65.8, −6.7). It showed peak activities in the left hemisphere and involved the fusiform gyrus (83.3%), declive (5.6%), inferior temporal gyrus (ITG, 5.6%), and MTG (5.6%). The fifth cluster was centred at the right fusiform gyrus (44.1, −58.7, −12). This cluster involved the fusiform gyrus only.

### 3.3. Contrast of Activation Maps Between Deaf and Hearing Individuals

**Figure 4:**
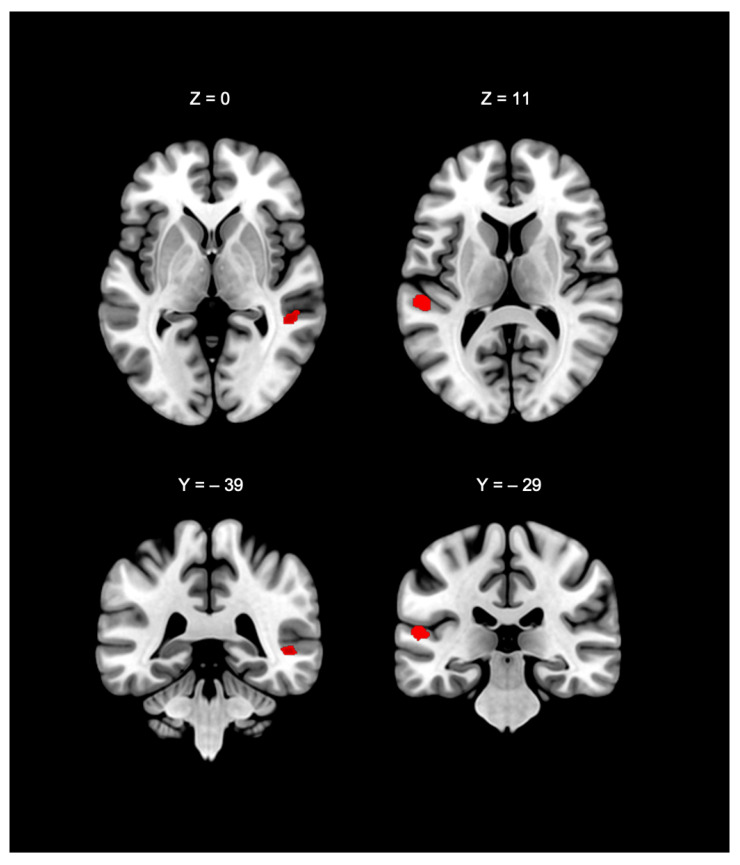
ALE cluster maps generated by Deaf > hearing contrast analysis. Red = regions showing a significant convergence of activation foci in deaf participants compared to hearing participants.

We conducted an analysis to contrast the spatial convergence of activation in deaf participants compared to hearing participants during nonverbal communication processing. Contrast analysis (see Table 4 and Figure 4) showed two significant clusters. The first cluster was centred at the left STG (−54.8, −29.9, 10.6), with peak activities in the left STG (78.6%) and transverse temporal gyrus (21.4%). The second cluster was centred at the right MTG (50, −39.5, −0.3). Given that the size of cluster 2 was small, the contrast statistical analysis did not involve a detailed composition of this cluster. Hence, only the location of the cluster centre was reported.

### 3.4. Jackknife Sensitivity Analysis

The Jackknife sensitivity analysis revealed that one of the two significant clusters identified in the ALE contrast analysis remained consistently present across all 14 simulations, regardless of which individual experiment was omitted. Specifically, cluster 1, with peaks in the STG, was observed in every simulation, whereas cluster 2 appeared in fewer than half of the simulations and was therefore considered unreliable. Accordingly, our discussion of the deaf > hearing contrast (Section 4.3) will focus on the role of the STG.

## 4. Discussion

We hypothesized that the auditory and visual cortices would show convergence of activation across studies during nonverbal communication processing in deaf adults compared to hearing adults, reflecting intra- and cross-modal plasticity following hearing deprivation.

### 4.1. Nonverbal Communication Processing in Deaf Adults

#### 4.1.1. Broca’s Area

Broca’s area, the posterior part of the IFG, was activated during nonverbal communication perception in deaf adults. It is long known for its role in language production regardless of modality [65]. Still, it showed convergence of activation even though none of the studies involved an active language production component.

The cluster in Broca’s area, by nonverbal communication observation, could be explained by the mirror neuron network [66]. Mirror neurons discharge in the execution of specific motor action and the observation of the same action [67]. Using a repetition suppression paradigm, Kilner and colleagues [66] showed IFG suppression when action observation was followed by action execution for the same action rather than a different action and when action execution was followed by action observation for the same action rather than a different action. They concluded that such a response pattern likely provides evidence of mirror neurons in human IFG. Here, we also observed the involvement of left IFG, particularly in Broca’s area, in response to nonverbal communication. A recent ALE meta-analysis investigating the functional neuroanatomy of sign language also found convergence in Broca’s area in sign language comprehension [38], which is consistent with our findings.

#### 4.1.2. Fusiform Gyrus

The convergence of the fusiform gyrus indicates facial expression processing in deaf adults. Given that sign language and speechreading involve observing the facial features as linguistic markers representing different lexical and syntactic structures [68], activities in the fusiform gyrus should be expected. The left-lateralized convergence in the fusiform gyrus may indicate the linguistic processing of facial features, consistent with previous findings of the left fusiform gyrus convergence for both emotional and linguistic facial expression [69]. Recognizing linguistic facial emotion requires identifying specific facial features, and the persistent brain activity associated with processing local facial features in deaf adults may thus contribute to the observed left lateralization within the fusiform.

### 4.2. Nonverbal Communication Processing in Hearing Adults

#### 4.2.1. Precentral Gyrus

Evidence from a clinical study suggests that the precentral gyrus plays an active role during speech production [70]. Damage to the left precentral gyrus is associated with apraxia of speech, characterized by articulatory distortion, slow rate speech, and lengthened segmentation of syllables [71]. However, in the present meta-analysis, none of the studies involved speech production tasks. Hence, convergence mass in the precentral gyrus for hearing participants might indicate other functions of the precentral gyrus. Indeed, a recent study investigating brain activation patterns during silent reading found a significant convergence in the precentral gyrus in a group of epileptic patients [72]. During intracranial electrophysiological recording, patients had to select animate nouns from a list of non-animate words, letter strings, and false-font stimuli. The evoked response of the precentral gyrus to linguistic stimuli was comparable to the activity around perisylvian regions. Therefore, the precentral gyrus is also involved in language perception, at least during silent reading. The precentral gyrus is also activated during audiovisual speech perception [73]. Watching and listening to video clips of the storyteller activated a network of frontal brain regions that includes the IFG, precentral gyrus and sulcus, postcentral gyrus, and cerebellum. However, listening to the audio-only or watching the visual-only version of the same video did not elicit the same level of convergence in these areas as in the audio-visual condition. This suggests that, in hearing individuals, language perception engages the precentral gyrus, which is associated with motor production, as demonstrated in a recent study [74].

#### 4.2.2. STG

STG is a part of the auditory ventral stream, a pathway that plays a role in auditory object formation and sound identification [75,76], particularly in tasks involving semantic judgement [57,63] and rhyming [13,53]. Additionally, the STG, particularly the posterior STG, is activated during audiovisual speech. Electrocorticography studies investigating the neural substrates of audiovisual speech processing have consistently found evidence of the STG’s role in multisensory processing [77,78], with greater activation in the anterior STG during audiovisual speech with clear audio, and a critical role of the posterior STG in audiovisual integration when the audio is degraded. Therefore, the activated clusters observed in studies with hearing participants likely reflect the process of object identification when audio-visual input is involved.

#### 4.2.3. Fusiform Gyrus

Activities in the bilateral fusiform gyrus of hearing individuals suggest facial expression processing, given that both sign language and speechreading involve observing the facial features as linguistic markers representing different lexical and syntactic structures [68]. This result is consistent with previous findings by McCullough et al. [69], the bilateral fusiform gyri are consistently activated for both emotional and linguistic facial expressions, indicating a uniform function of facial processing in hearing non-signers. In contrast, deaf signers show left-lateralized convergence in the fusiform gyrus for emotional and linguistic facial expressions. Given that fusiform gyrus convergence was comparable in deaf and hearing individuals, the bilateral fusiform gyrus is likely involved in early analysis of facial features unrelated to the knowledge of sign languages. The fusiform gyrus is also associated with face perception in speech reading tasks [58,60]. Both types of nonverbal communications consistently activated the fusiform gyrus in hearing participants when performing tasks with nonverbal communication stimuli, most involving face perception. Importantly, studies with deaf participants did not show consistent convergence in the occipital lobe responsible for visual perception. This may reflect the cross-modal plasticity that leads to preferential access to the auditory speech areas of the left STG when viewing speech-like oral actions.

#### 4.2.4. IPL

Only the right IPL was active during nonverbal communication in hearing individuals. Singh-Curry and Husain [79] reviewed lesion, functional imaging, and electrophysiological studies and proposed that the right IPL maintains attentive control of the task at hand (e.g., holding target characteristics in mind to direct subsequent actions) and reacting to salient environmental stimuli. In contrast, the left IPL is part of a network for semantic processing [80,81]. Convergence mass in the right but not left IPL may reflect that visual information was not considered linguistic or semantic. For instance, certain hearing participants had limited exposure to speechreading [54,55,58,63,82,83] and would have difficulty decoding lip movements into words. Given the mixed experience in processing nonverbal stimuli, convergence of IPL in hearing individuals may reflect a general state of sustained attention to the ongoing task.

### 4.3. Contrast of Deaf > Hearing Individuals

The contrast analysis did not reveal a consistent intramodal convergence in the visual cortex in deaf compared to hearing individuals. The lack of group differences in the visual cortex during nonverbal communication can be explained by the type of visual stimuli used in the studies. This meta-analysis only includes studies that used visual linguistic stimuli and excludes those that used pure shapes. Past studies found greater intramodal visual cortex activation in CI and generally employed non-linguistic related shapes like high-contrast sinusoidal concentric gratings [20,84]. Since those shapes do not contain language-relevant content and are purely assessing visual perception, the auditory cortex that processes language, regardless of modality, might not be as activated as when processing language-relevant stimuli. Nonetheless, evidence for intra-modal reorganization of the visual cortex (especially in postlingually deaf people without a CI) is sparse, and more work is needed to segregate the intra-modal activation into different types of visual stimuli.

#### STG

The contrast analysis found greater convergence in the left STG of deaf adults than in hearing adults. Consistent with our hypothesis of greater cross-modal plasticity in hearing loss, nonverbal communication processing activates the auditory cortices of deaf adults more than those of hearing individuals. Previous investigations into language processing in deaf adults have also shown STG activation. For example, Twomey et al. [62] conducted a study with deaf signer participants who performed a BSL phonological task. This task required them to determine whether two presented pictures shared the same BSL handshape and hand location, two phonological parameters in sign language [85]. Visual semantic judgement and discrimination tasks were also administered to compare task difficulties. The study revealed bilateral STG activations, with the right STG being more task-independent and consistently activating regardless of task type. In contrast, the left STG was task-dependent, activating only in the BSL phonological task that required visual-spatial processing.

This meta-analysis provides converging evidence on the role of the STG in visual processing, particularly within the language processing domain. Convergence in left STG could relate to a greater demand for visual-spatial processing in deaf adults. Considering that nine out of 14 studies in the current analysis involve sign language processing, and six of the nine sign language studies consist of tasks requiring active behavioural responses to the semantic level of sign languages, left STG may contribute to the visual-spatial processing of stimuli. For example, some tasks required participants to identify semantically anomalous BSL sentences [6,62], while other studies asked participants to judge whether the signed words were actual words or belonged to a specific category [61,63]. All these tasks demanded greater effort in processing visually presented hand gestures and facial expressions, reflecting higher task demand. In addition to sign language, other non-verbal communication modalities also involve STG. For instance, more proficient deaf readers engaged the left STG to a greater extent, mirroring the pattern observed in hearing readers, unlike their less proficient peers [10]. When presented with silently spoken words, deaf native signers show greater activation in STG than hearing, even though both groups were able to speechread [86]. Taken together, these findings highlight the STG as a key hub supporting non-verbal language processing across multiple modalities in deaf individuals.

Critically, the results also revealed more convergence mass (i.e., cluster size) in the left hemisphere compared to the right. This result could be partially attributed to the left-lateralized nature of language, including visually presented language [87]. However, this asymmetry could also result from the higher task demand in those studies. For instance, determining whether a sentence was semantically anomalous required encoding each signed word, retrieving word meanings, combining words into sentences, and eventually deciding whether the sentences followed logical rules. Consequently, the left hemisphere perisylvian language areas were recruited to facilitate this complex process [88].

### 4.4. Limitations

The present study included only pre-lingually deaf participants to minimize the confounding of the age of deafness. However, as the population of post-lingual hearing loss is growing, it is also important to understand this population’s functional anatomy of nonverbal communication. Although both pre-lingual deaf and post-lingual deaf adults experienced profound hearing loss, it is crucial to acknowledge that these two populations exhibit distinct patterns of activation when visually perceiving language. After exposure to spoken language before auditory deprivation, postlingually deaf individuals likely undergo a sensitive period for cortical auditory development during childhood, wherein the auditory pathway demonstrates maximum plasticity. Throughout this critical period, extrinsic sensory stimulation, such as spoken language input, can profoundly influence the developmental and organizational aspects of the auditory cortex [89,90,91]. The early successful reorganization in post-lingually deafened individuals may result in an auditory system that more closely approximates the regular auditory system than that of pre-lingually deafened individuals. Consequently, the heterogeneity in participant characteristics could introduce confounding factors into the convergence patterns. Additionally, there could be preferential differences in nonverbal communication between pre- and post-lingually deaf adults. For example, given the exposure to auditory language, post-lingually deaf adults may find lip-reading/speech-reading easier to comprehend than sign language. Subsequent research could investigate whether pre-lingually and post-lingually deafened individuals display differential cross-modal plasticity with sufficient statistical power.

Given the focus of this paper and the inclusion criteria applied, we acknowledge that the number of studies included in this ALE meta-analysis falls below the recommended threshold for such analyses [49]. To address this limitation, we adopted a more conservative methodological approach, employing a greater number of permutations, stricter mask sizes, and thresholding values when conducting the ALE.

There is currently no clear consensus on what constitutes nonverbal communication. Although we broadly define non-auditory linguistic stimuli as nonverbal communication, we recognize that this category encompasses heterogeneous modalities. Written language is considered a direct “translation” of spoken languages, whereas sign language is thought of as a natural language on its own with a different modality [65,92]. Nevertheless, the overarching aim of this study is to identify the common neural networks that support linguistic processing across diverse modalities in individuals with auditory deprivation. Future research could aim to disentangle the modality-specific contributions to these networks, examining how tasks such as speechreading, sign language, and written languages differentially engage neural circuits in both deaf and hearing populations. Such studies would provide a more nuanced understanding of cross-modal plasticity and the functional role of each nonverbal communication modality.

### 4.5. Implications

Identifying consistent brain regions involved in visual language processing offers valuable insights for customizing therapeutic interventions. Healthcare professionals can develop targeted interventions to enhance specific neural pathways associated with nonverbal communication in deaf individuals. While some previous views have suggested refraining from using visual communication before cochlear implantation to mitigate potential adverse effects on the auditory cortex, it is impractical for deaf individuals to abstain from employing visual languages for communication, and more recent work has shown that cross-modal plasticity may benefit real-world speech perception (reviewed in [21]). Subsequent studies should explore whether areas activated during visual language perception negatively correlate with CI outcomes. Based on such findings, interventions could be tailored to optimize the quality of life and CI outcomes for deaf individuals by focusing on specific brain regions.

## 5. Conclusions

Because hearing loss limits spoken language processing, deaf individuals often use non-verbal communication as a primary means of interaction. Such visual linguistic reliance on speechreading, sign and written languages induces cross-modal plasticity, resulting in distinct sensory processing in deaf compared with hearing individuals. By conducting a coordinate-based meta-analysis, this study displayed the neural substrates of visual language processing in deaf adults. Specifically, deaf participants consistently exhibit more consistent convergence in left STG than hearing participants. These areas are related to auditory processing and language understanding, indicating neuroplastic changes in visual language processing in response to the lack of auditory input. Importantly, understanding these neural adaptations has implications for therapeutic intervention design and clinical decision-making. Knowledge of how visual language is reorganized in the deaf brain can help optimize rehabilitation strategies, inform expectations for cochlear implant outcomes, and guide the development of interventions that account for pre-existing cross-modal reorganization. Future studies are needed to investigate whether this cross-modal plasticity relates to CI outcomes and assess the impact of hearing restoration on the visual-induced activations in the temporal lobe.

## Figures and Tables

**Table 1:** Neuroimaging studies included in the meta-analysis.

Study	Task	Contrast	Participants Demographics	Foci (Deaf, Hearing)	Coordinate Space
[4]	Speechreading: watched the speech patterns produced by the model and made a response when the model was seen to be saying ‘yes’.	Deaf: speechreading vs. rest; hearing: speechreading vs. rest	Deaf participants: *n* = 13, mean age (SD): 27.4 (7.6). Hearing participants: *n* = 13, mean age (SD): 29.4 (6.15).	12, 12	Talairach
[7]	British Sign Language (BSL) Task: identified semantically anomalous BSL sentences. Baseline = rest.	deaf signers: BSL vs. baseline; hearing signers: BSL vs. baseline	Deaf signers: *n* = 8, mean age (range): 30.5 (18–48), all acquired BSL from deaf parents. Hearing signers: *n* = 8: mean age (range): 32.8 (20–51); all were native signers; six of the hearing native signers were employed as a BSL interpreter or communicator.	8, 16	MNI
[53]	Visual rhyming task (VRT): judged whether two French words rhyme. Baseline condition: identical strings judgement task.	Deaf: VRT vs. baseline; Hearing: VRT vs. baseline	Deaf participants: *n* = 4, mean age: 28.25. All wore hearing aids. Hearing participants: *n* = 15, mean age: 26.	17, 3	MNI
[54]	For deaf participants: Passively viewed videos of (a) cued speech: oral + manual (CSLM); (b) cued speech manual, (c) cued speech oral (lip reading). For Hearing: audio-visual speech, auditory speech, visual speech: lipreading. Baseline = still.	Deaf: CSLM vs. baseline; Hearing: audio-visual speech vs. baseline	Deaf participants: *n* = 14 (13 were congenitally deaf), mean age (range): 25 (18–33). All were fitted with hearing aids between 6 months and 2 years of age. Hearing participants: *n* = 15, mean age (range) *: 25.2 (20–37).	6, 3	MNI
[55]	Video watching: watched videos of pantomimes (e.g., peeling an imaginary banana) and action verbs in American Sign Language (ASL). Baseline = stare at a fixation cross.	Deaf: ASL vs. baseline; Hearing: ASL vs. baseline	Deaf participants: *n* = 14, mean age (range): 22.3 (19–43); all were native signers. Hearing participants: *n* = 14, mean age (range): 24.3 (22–29); all had no knowledge of a signed language.	6, 25	MNI
[56]	Silent video watching: watched a video in which a translator told a story using German Sign Language (GSL).	Deaf: sign language watching vs. rest; Hearing: sign language watching vs. rest	Deaf participants: *n* = 12, mean age: 46, Mean age on losing hearing (years): 2.2; all were proficient in GSL (good to excellent). Hearing participants: *n* = 12, mean age: 32; none of them knew GSL at all.	6, 2	Talairach
[57]	Sign language observation for deaf participants: watched videos of common objects lexicalized in GSL, Sound listening for Hearing: listened to sounds from animals and artificial objects.	Deaf: sign language observation vs. baseline; Hearing: sound listening vs. baseline	Deaf participants: *n* = 16, mean age (range): 43.7 (33–67), mean age on losing hearing (months): 11.38, all were native GSL signers. Hearing participants: *n* = 18, mean age (range): 41.3 (19–68); none of them know GSL at all.	67, 39	MNI
[58]	Silent speechreading: watched the speaker in the video and covertly repeated the numbers. Baseline: counted gurning faces.	Deaf: speechreading vs. baseline; Hearing: speechreading vs. baseline	Deaf participants: *n* = 6, mean age (range): 30 (29–38). Hearing participants: *n* = 7, mean age (range): 29 (21–55).	8, 7	Talairach
[59]	Participants watched signed BSL sentences or audio-visual English translations of the same sentences and chose semantically anomalous BSL sentences. Baseline = rest.	Deaf: BSL vs. baseline; Hearing: BSL vs. baseline	Deaf participants: *n* = 9, mean age: 30.4; all were native signers. Hearing participants: *n* = 9, mean age: 32.67; all were native signers.	11, 11	Talairach
[60]	Attended alternating blocks of four different visual stimuli: checkerboard, words, sign language, and lip-reading. Baseline = rest.	Both groups: words vs. baseline; sign language vs. baseline; lip-reading vs. baseline	Deaf participants: *n* = 34, mean age: 20.8, mean age on losing hearing (years): 1.6; all were proficient signers. Fifteen deaf participants used hearing aids. Hearing participants: *n* = 15, mean age: 20.3; all had no knowledge of sign language.	16, 3	MNI
[61]	Sign-word discrimination task: decide whether hand movements constitute a real word.	Deaf: sign-word discrimination vs. rest; Hearing: sign-word discrimination vs. rest	Deaf participants: *n* = 7, mean age ± SD: 31.6 ± 7.1, Age on losing hearing (years): <3; All were fluent in sign language. Hearing participants: *n* = 19, mean age ± SD: 10.7. All were fluent in sign language.	24, 25	Talairach
[62]	BSL sentence judgement: determined whether a BSL sentence contained a semantic anomaly. Baseline = fixation.	Deaf: BSL vs. baseline; Hearing: BSL vs. baseline	Deaf signers: *n* = 15, mean age (range): 34.25 (23.5–59); all were native signers. Five wore hearing aids. Hearing signers: *n* = 14, mean age (range): 34.5 (20.25–60), all were native signers.	9, 4	MNI
[63]	BSL sign task: pressed a button when the item signed was an animal; baseline: responded when the fixation cross went red.	Deaf signers: BSL vs. baseline; hearing signers: BSL vs. baseline	Deaf signers: *n* = 13, mean age (range): 27.4 (18.9–49.25); all were native signers. Hearing non-signers: *n* = 13, mean age (range): 29.7 (18.9–43.3), all had no prior exposure to sign language.	2, 2	Talairach
[64]	Semantic judgement reading task: judged whether two-word, grammatically correct phrases were meaningful or nonsensical. Baseline: searched visually for hashtags (#) embedded in two-word consonant strings	Both groups: semantic judgement reading task vs. baseline	Deaf participants: *n* = 12, mean age ± SD: 27.4 ±4.4. Eight used hearing aids. Hearing participants: *n* = 20, mean age ± SD: 23.7 ± 1.4.	10, 2	MNI

* For studies that did not provide the standard deviation (SD) of age, the age range was reported instead.

**Table 2:** Brain areas consistently activated as a function of nonverbal communication in deaf people.

Cluster #	Cluster Size	Anatomical Area	ALE Value	*p* Value	z-Score	MNI Coordinates (x y z)			# Studies/ Cluster *
1	3112 mm^3^	Left superior temporal gyrus, BA41	0.016	<0.001	4.65	−56	−22	8	6
		Left superior temporal gyrus, BA22	0.015	<0.001	4.43	−60	−8	2	
		Left superior temporal gyrus, BA41	0.015	<0.001	4.39	−56	−28	10	
		Left superior temporal gyrus, BA41	0.014	<0.001	4.08	−46	−34	10	
		Left superior temporal gyrus, BA22	0.013	<0.001	3.92	−64	−18	−2	
2	1912 mm^3^	Right superior temporal gyrus, BA41	0.013	<0.001	3.98	54	−24	4	8
		Right superior temporal gyrus, BA21	0.013	<0.001	3.9	54	−24	−6	
		Right sub-gyral, BA37	0.013	<0.001	3.89	50	−40	−4	
		Right superior temporal gyrus, BA22	0.012	<0.001	3.72	52	−34	4	
3	1512 mm^3^	Left inferior frontal gyrus, BA9	0.015	<0.001	4.45	−46	12	28	7
		Left inferior frontal gyrus, BA45	0.014	<0.001	4.13	−48	18	14	
4	776 mm^3^	Left middle temporal gyrus, BA37	0.010	<0.001	3.38	−54	−60	2	5
5	728 mm^3^	Left fusiform gyrus, BA37	0.014	<0.001	4.32	−42	−62	−14	3

* # Studies/Cluster = number of studies contributing to this cluster.

**Table 3:** Brain areas consistently activated as a function of nonverbal communication in hearing individuals.

Cluster #	Cluster Size	Anatomical Area	ALE Value	*p* Value	z-Score	MNI Coordinates (x y z)			# Studies/ Cluster *
1	2088 mm^3^	Left superior temporal gyrus, BA22	0.022	<0.001	5.55	−64	−18	2	5
2	1200 mm^3^	Left precentral gyrus, BA4	0.015	<0.001	4.41	−48	−4	46	5
		Left middle frontal gyrus, BA6	0.012	<0.001	3.89	−44	6	38	
3	1184 mm^3^	Right inferior parietal lobule, BA39	0.019	<0.001	5.08	34	−48	42	5
4	1136 mm^3^	Left fusiform gyrus, BA37	0.015	<0.001	4.29	−44	−66	−8	4
5	880 mm^3^	Right fusiform gyrus, BA37	0.020	<0.001	5.29	44	−60	−12	4

* # Studies/Cluster = number of studies contributing to this cluster.

**Table 4:** Results of the contrast analysis (deaf > hearing).

Cluster #	Cluster Size	Anatomical Area	*p* Value	z-Score	MNI Coordinates (x, y, z)		
1	584 mm^3^	Left superior temporal gyrus, BA41	0.0159	2.15	−56	−32	12
		Left superior temporal gyrus, BA41	0.0178	2.1	−54	−28	10
2	192 mm^3^	Right middle temporal gyrus, BA21	0.0167	2.09	52	−38	2

## Data Availability

The original contributions presented in the study are included in the article/Supplementary Materials. Further inquiries can be directed to the corresponding author.

## Supplementary Materials

The following supporting information can be downloaded at: https://www.mdpi.com/article/10.3390/brainsci15121299/s1, File S1: PRISMA 2020 Checklist.
