# Supplementary material for: Nonverbal Communication Processing in Deaf Adults: An Activation Likelihood Estimation Meta-Analysis
Source: Brain Sci. 2025 Nov 30;15(12):1299. doi: 10.3390/brainsci15121299 (PMC12730276; doi:10.3390/brainsci15121299)
Supplement: Supplementary file 1 [file brainsci-15-01299-s001.zip › brainsci-3885911-supplementary.pdf]

PRISMA 2020 Checklist

Section and Topic	Item #	Checklist item	Location (page) where item is reported
**TITLE**			
Title	1	Visual language processing in deaf individuals: An Activation likelihood estimation meta-analysis	1
**ABSTRACT**			
Abstract	2	Background: Hearing loss affects spoken language processing and leads to cortical reorganization in sensory systems. Although significant research has explored cross-modal plasticity in visual language processing within the temporal lobe, there is a need for a comprehensive integration of brain activation patterns across deaf individuals.<br>Methods: We conducted an activation likelihood estimation on 14 neuroimaging studies to identify cortical regions with greater activation during visual language processing in hearing impaired individuals compared to hearing individuals.<br>Results: Hearing impaired individuals did not show greater intramodal activation in the visual cortex but had increased activation in the bilateral superior and middle temporal gyri.<br>Conclusion: Our meta-analysis showed stronger cross-modal activation in the auditory cortex in individual with hearing impairment than in those with normal hearing.	1
**INTRODUCTION**			
Rationale	3	Hearing loss affects spoken language processing and leads to cortical reorganization in sensory systems. Although significant research has explored cross-modal plasticity in visual language processing within the temporal lobe, there is a need for a comprehensive integration of brain activation patterns across deaf individuals.	8
Objectives	4	The primary goal of this study is to identify the neural correlates of visual language perception in deaf individual, shedding light on the reorganization of the brain network following auditory deprivation.	8
**METHODS**			
Eligibility criteria	5	Articles were included if they met the following criteria: (a) language-related materials are presented visually (low-level stimuli like checkerboards and simple shapes were excluded); (b) a behavioural task performed during the scanning; (c) a control or baseline condition; (d) whole-brain analysis from fMRI or positron emission tomography (PET) on 3D coordinates in either Talairach (33) or Montreal Neurological Institute (MNI) standardized space; (e) deaf individuals, and hearing individual, all of which had no psychiatric or neurological disorders, or brain abnormalities (e.g., stroke, tumor); (f) congenitally deaf or prelingually deafened. Studies meeting these selection criteria used a variety of stimuli and tasks, such as speechreading tasks, phonological processing tasks, and sign language judgement tasks.	11
Information sources	6	A search was conducted in PubMed, Medline, and ProQuest. The search included studies published in English peer-reviewed journals as of August 2025.	8
Search strategy	7	A search was conducted in PubMed, Medline, and ProQuest using the following terms: “hearing loss”, “deafness”, “visual language perception”, “lip reading”, “neuroplasticity”, “brain reorganization”, crossed with “fMRI”, “PET”, “functional magnetic resonance imaging”, “positron emission tomography”, and “neuroimaging”.	9
Selection process	8	Title and abstract of each record were screened based on the eligibility criteria in item 5. Full texts of potentially eligible articles were then retrieved and screened.	9
Data collection process	9	Title and abstract of each record were screened based on the eligibility criteria in item 5. Full texts of potentially eligible articles were then retrieved and screened.	9
Data items	10a	Extracted the first author’s name, publication year, sample size from the final included studies.	9
	10b	Extracted foci, participants’ demographics, neuroimaging techniques, and methods used from the final included studies.	9
Study risk of bias assessment	11	One reviewer extracted eligible studies. Two reviewers assessed each extracted study independently.	9

PRISMA 2020 Checklist

Section and Topic	Item #	Checklist item	Location (page) where item is reported
Effect measures	12	Foci reported for activated brain regions during visual language perception were extracted.	10
Synthesis methods	13a	Articles were included if they met the following criteria: (a) language-related materials are presented visually (low-level stimuli like checkerboards and simple shapes were excluded); (b) a behavioural task performed during the scanning; (c) a control or baseline condition; (d) whole-brain analysis from fMRI or positron emission tomography (PET) on 3D coordinates in either Talairach (33) or Montreal Neurological Institute (MNI) standardized space; (e) deaf individuals, and hearing individual, all of which had no psychiatric or neurological disorders, or brain abnormalities (e.g., stroke, tumor); (f) congenitally deaf or prelingually deafened. Studies meeting these selection criteria used a variety of stimuli and tasks, such as speechreading tasks, phonological processing tasks, and sign language judgement tasks.	10
	13b	NA	
	13c	GingerALE was used for activation likelihood estimation (ALE) analysis; 2D cluster ALE images and 3D image were created using MRIcroGL	10
	13d	Coordinate-based quantitative meta-analyses of neuroimaging results were performed using the GingerALE software (version 3.0.2) that is available on the BrainMap website (http://brainmap.org/ale/index.html). Talairach coordinates were converted to MNI space using the nonlinear registration before being entered into the analysis. Based on coordinates from the included articles, GingerALE generates a brain activation map and applies a permutation test to assess the statistical reliability of the group mean activation.	10
	13e	NA	
	13f	NA	
Reporting bias assessment	14	NA	
Certainty assessment	15	NA	
**RESULTS**			
Study selection	16a	A thorough exploration across all databases identified 152 studies, as depicted in the PRISMA flowchart (32) in Fig 1. Among these, 138 papers were excluded after screening titles or abstracts. The primary reasons for exclusion included the absence of activation coordinates, lack of a behavioural task related to visual language perception, absence of a hearing control group, or the absence of prelingual deaf participants. Following this screening process, 14 papers were deemed suitable for coordinate-based meta-analysis, encompassing 177 deaf participants and 192 NH participants.	11
	16b	A thorough exploration across all databases identified 152 studies, as depicted in the PRISMA flowchart (32) in Fig 1. Among these, 138 papers were excluded after screening titles or abstracts. The primary reasons for exclusion included the absence of activation coordinates, lack of a behavioural task related to visual language perception, absence of a hearing control group, or the absence of prelingual deaf participants. Following this screening process, 14 papers were deemed suitable for coordinate-based meta-analysis, encompassing 177 deaf participants and 192 NH participants.	11
Study characteristics	17	See Table 1	13
Risk of bias in studies	18	NA	
Results of individual studies	19	See Table 1 and supporting document	13
Results of syntheses	20a	NA	
	20b	See Table 2, 3 and 4	21, 24, 26

Section and Topic	Item #	Checklist item	Location (page) where item is reported
	20c	NA	
	20d	NA	
Reporting biases	21	NA	
Certainty of evidence	22	NA	
**DISCUSSION**			
Discussion	23a	Contrary to expectations, deaf individuals did not show greater intramodal activation in the visual cortex but had increased activation in the bilateral superior and middle temporal gyri, supporting stronger cross-modal activation in the auditory cortex.	27
	23b	The present study included only prelingually deaf participants to minimize the confounding of the age of deafness. However, as the population of postlingual hearing loss is growing, it is also important to understand this population's functional anatomy of visual language perception. Although both prelingual deaf and postlingual deafened individuals all experienced profound hearing loss, it is crucial to acknowledge that these two populations exhibit distinct patterns of activation when visually perceiving language.	35
	23c	Due to the inclusion and exclusion criteria, number of studies included is limited.	35
	23d	Future research investigating the relationship between neuroplasticity and speech perception outcomes following auditory restoration with neuroprostheses like cochlear implants would define the preconditions for successful hearing restoration.	35
**OTHER INFORMATION**			
Registration and protocol	24a	NA	
	24b	NA	
	24c	NA	
Support	25	NA	
Competing interests	26	The authors declare that they have no conflict of interest.	
Availability of data, code and other materials	27	Foci used for the ALE analysis are available in the supporting information document.	

From: Page MJ, McKenzie JE, Bossuyt PM, Boutron I, Hoffmann TC, Mulrow CD, et al. The PRISMA 2020 statement: an updated guideline for reporting systematic reviews. BMJ 2021;372:n71. doi: 10.1136/bmj.n71

Foci extracted from 14 papers

The following are foci included in the activation likelihood estimation meta-analysis. Note that they are formatted according to GingerAle's requirements.

Foci of Visual Language Perception in Deaf Individuals

// Reference=MNI
// Lambertz et al., 2005
// Subjects=12

59	-17	1
67	-20	-6
62	-12	0
-62	-8	-1
64	-23	-6
57	14	19

// Trumpp & Kiefer, 2018
// Subjects=16

-32	-20	0
4	-56	-14
-12	-24	-2
-36	-58	-8
-30	-14	10
10	-52	-14
36	-42	44
-40	-18	-10
-12	-60	-16
-40	-64	-16
4	-70	18
42	-62	-14
16	0	8
56	12	0
-56	-30	14
58	12	22
-42	-70	-10
54	-34	8
50	12	34
12	-10	10
36	-2	8
-34	34	14
-36	50	12
32	48	-4
6	38	10
64	-34	16
56	-22	-8
54	4	-18
24	-98	6
52	-72	-2
2	-86	-2
-48	12	28
-12	-104	12
18	-96	-8
-32	-96	-6

54	-66	-2
10	-98	-4
12	-100	2
-56	-62	2
-44	-72	-14
-48	-80	0
44	-74	-12
28	-96	-12
-50	0	-20
-53	36	12
-42	-58	-24
-58	-24	-12
-58	-4	-12
-46	0	56
-36	-88	-16
52	42	18
54	18	22
50	22	16
34	-62	58
40	-48	50
22	-26	-2
28	-14	-10
0	10	60
-6	2	66
-4	24	52
-20	-26	-6
44	0	60
50	6	54
48	8	42
-40	-46	54
16	-6	54
22	0	-18

// Sadato et al., 2005
// Subjects=7

-60	-17	2
-66	-19	1
-1	1	74
8	21	50
-50	16	20
-36	-3	54
-31	32	-6
50	34	-2
47	46	15
35	-60	53
39	-51	62
-26	-66	60
-26	-51	52
-30	-58	57
-20	-81	43
54	-58	-6

-64	-17	-2
66	-8	-6
-49	-71	-1
-52	-56	2
-35	-62	-39
-66	-19	1
-61	-8	1
-57	-24	8

// Capek et al., 2008
// Subjects=13

54	-5	-9
-57	-22	8
48	-6	42
-5	13	47
-12	-43	-40
50	-25	-9
38	18	4
49	-19	11
49	22	25
-1	15	36
-29	-8	58
32	-4	57

// Emmorey et al., 2010
// Subjects=14

-12	66	3
-48	18	12
-57	-48	-1
45	-43	2
-42	-60	-12
50	-65	-12

// Aparicio et al., 2009
// Subjects=4

-46	0	30
-50	24	24
-60	-46	8
-54	-8	38
-52	20	20
-60	-38	0
-6	8	58
-34	-74	42
30	-62	-36
-54	-10	18
50	-2	44
-44	-12	42
-42	14	28
-44	8	34
-40	-48	50
-46	-56	-14

-52 22 0

// MacSweeney et al., 2004
// Subjects=8
-50 -49 -1
51 -41 -4
43 23 12
-44 12 30
-45 39 -4
10 30 31
17 10 5
41 -59 -33

// MacSweeney, Calvert et al., 2002
// Subjects=6
3 -47 25
10 41 -6
11 -91 13
3 -62 46
-10 -61 26
2 -83 5
-3 -65 6
-10 -94 -15

// Que et al., 2018
// Subjects=34
66 -27 15
69 -24 9
-48 -33 12
60 -30 15
54 -24 6
66 -30 12
-66 -30 6
-63 -30 6
54 -24 6
66 -24 12
-66 -30 6

// Twomey et al., 2020
// Subjects=15
-45 -34 8
-54 -31 8
-54 -19 5
-51 -22 2
-60 -10 5
-63 -19 -1
-60 -34 -11
54 -25 -1
63 -7 -7

// MacSweeney, Woll et al., 2002
// Subjects=9
-47	-51	1
51	-41	-6
-56	-14	-28
-44	19	14
45	21	7
2	27	40
-24	-71	37
49	-48	32
-36	-41	42
17	12	4
-19	7	-1

// Aparicio et al., 2017
// Subjects=14
-54	-74	4
52	-68	2
-50	32	2
52	-34	2
-56	-4	48
-50	-48	-24

// Waters et al., 2007
// Subjects=13
-49	-63	-14
44	-55	-18
-40	19	26
47	0	40

// Kotowicz et al., 2024
// Subjects=12
−62	−50	6
−38	6	30
−34	36	12
−32	26	2
−8	−80	−20
−18	−92	−4
42	30	24
6	16	48
34	24	−4
10	−74	−24

Foci of Visual Language Perception in NH

// Reference=MNI
// Lambertz et al., 2005
// Subjects=12
67	-37	5
53	-76	-2

// Trumpp & Kiefer, 2018

// Subjects=18

-30	-20	2
18	-10	14
4	-54	-14
-36	-56	-14
-36	16	-6
-18	8	2
-18	-30	-2
-34	-10	-4
16	0	8
26	-42	-18
-12	6	10
12	8	6
-14	-20	-4
48	-70	-4
42	-60	-14
22	-28	-6
-46	-68	0
34	-10	-8
44	-36	46
36	-46	42
28	-60	40
-2	22	36
-4	10	28
-2	-4	64
-46	-24	16
-44	-32	40
-50	-32	50
-26	-14	60
6	-42	54
16	-34	50
-32	42	26
-32	36	12
-58	-42	22
-62	-18	2
-60	-6	-4
-50	-22	2
62	-28	2
48	-30	4
60	-12	2

// Sadato et al., 2005
// Subjects=19

-48	18	-9
-50	12	36
-45	6	52
-49	-4	45
-3	-3	72
-5	11	72
-66	-15	2
-59	-1	-11

-64 -32 2
66 -23 -3
43 9 32
42 -4 61
-3 1 74
-5 17 58
-41 12 34
-28 0 69
-37 32 16
29 -66 53
37 -53 62
-30 -60 57
-47 -36 42
46 -60 -13
29 -77 24
-46 -66 -15
-56 -62 -3

// Capek et al., 2008
// Subjects=13
45 -29 -6
-45 -63 -6
-33 -55 46
35 -51 43
-48 -9 46
42 11 25
4 1 55
34 -45 11
11 -83 -14
-1 -34 23
-5 -12 32
53 -16 30

// Emmorey et al., 2010
// Subjects=14
-12 51 48
15 48 48
-60 21 2
51 22 36
-48 29 -10
30 36 -8
-45 3 39
54 9 41
6 19 6
-18 -22 13
-21 -36 2
-3 -45 32
-63 -48 0
61 -45 5
-49 -78 -8
44 -63 5

-42	-47	54
33	-46	48
-51	-66	39
45	-66	21
-39	-69	53
34	-58	53
-4	-75	25
2	-75	26
-36	-78	-36

// Aparicio et al., 2009
// Subjects=15

-46	20	26
-6	6	62
6	-92	0
-44	-2	48

// MacSweeney et al., 2004
// Subjects=8

-49	-56	-1
47	-52	-9
44	-39	-26
-44	18	18
48	19	14
-37	-50	43
-22	-74	39
22	-82	21
46	-52	-8
-40	-65	-1
46	23	14
-44	7	38
6	33	38
34	57	-1
-26	-51	44
34	-52	39

// MacSweeney, Calvert et al., 2002
// Subjects=7

63	-18	-14
-60	-16	-6
-66	-36	-15
-63	-25	4
4	15	38
-3	-89	0
-58	-34	0

// Que et al., 2018
// Subjects=15

54	-24	6
66	-30	12
-69	-27	6

// Twomey et al., 2020
// Subjects=14
-45 -13 5
-57 -19 8
-60 -7 5
48 -13 -1

// MacSweeney, Woll et al., 2002
// Subjects=9
-44 -55 10
49 -45 -11
-42 14 20
47 18 20
43 28 -17
2 30 36
32 -47 36
17 9 7
-14 2 9
27 -85 -13
25 -80 23

// Aparicio et al., 2017
// Subjects=15
-64 -20 2
62 -22 6
-50 28 14

// Waters et al., 2007
// Subjects=13
-44 -67 -10
43 -59 -10
43 −19 46

// Kotowicz et al., 2024
// Subjects=20
-58 -42 4
-50 30 2
